# Impacts of uncertainties in European gridded precipitation observations on regional climate analysis

**DOI:** 10.1002/joc.4706

**Published:** 2016-03-20

**Authors:** Andreas F. Prein, Andreas Gobiet

**Affiliations:** ^1^ MMM: Mesoscale & Microscale Meteorology Laboratory and Research Applications LaboratoryNational Center for Atmospheric Research (NCAR)BoulderCOUSA; ^2^ Wegener Center for Climate and Global ChangeUniversity of GrazGrazAustria; ^3^ Avalanche warning serviceCentral Institute for Meteorology and Geodynamics (ZAMG)GrazAustria

**Keywords:** observation uncertainties, precipitation, undercatch correction, climate models, high resolution, EURO‐CORDEX, extremes

## Abstract

Gridded precipitation data sets are frequently used to evaluate climate models or to remove model output biases. Although precipitation data are error prone due to the high spatio‐temporal variability of precipitation and due to considerable measurement errors, relatively few attempts have been made to account for observational uncertainty in model evaluation or in bias correction studies. In this study, we compare three types of European daily data sets featuring two Pan‐European data sets and a set that combines eight very high‐resolution station‐based regional data sets. Furthermore, we investigate seven widely used, larger scale global data sets. Our results demonstrate that the differences between these data sets have the same magnitude as precipitation errors found in regional climate models. Therefore, including observational uncertainties is essential for climate studies, climate model evaluation, and statistical post‐processing. Following our results, we suggest the following guidelines for regional precipitation assessments. (1) Include multiple observational data sets from different sources (e.g. station, satellite, reanalysis based) to estimate observational uncertainties. (2) Use data sets with high station densities to minimize the effect of precipitation undersampling (may induce about 60% error in data sparse regions). The information content of a gridded data set is mainly related to its underlying station density and not to its grid spacing. (3) Consider undercatch errors of up to 80% in high latitudes and mountainous regions. (4) Analyses of small‐scale features and extremes are especially uncertain in gridded data sets. For higher confidence, use climate‐mean and larger scale statistics. In conclusion, neglecting observational uncertainties potentially misguides climate model development and can severely affect the results of climate change impact assessments.

## 1 Introduction

Precipitation is a key parameter in the climate system and it is important for ecosystems, agriculture, water supply, or hydroelectric power production. Observing precipitation is challenging because it is highly variable in space and time Bacchi and Kottegoda (1995) and subject to substantial measurement errors. The latter mainly depend on the type and intensity of precipitation, the type of gauge employed, and on wind speed (Sevruk and Hamon, 1984).

One error that can be dominant in high latitudes and mountainous regions is precipitation undercatch. Undercatch errors for rain typically are between 3 and 20% and for snow they can be up to 40% or even 80% (in the case of non‐shielded gauges) (Førland and Institutt, 1996; Goodison *et al.*, 1997) The application of undercatch correction can modulate measured precipitation trends if they are related to lower undercatch errors due to more liquid precipitation in warmer climates (Førland and Hanssen‐Bauer, 2000).

An additional error source is the sampling error that mainly depends on the station density. For a given number of stations, the sampling error depends on the spatial variability of precipitation that is influenced by the orography, season, temporal resolution, and type of precipitation (convective, stratiform) (e.g. Schneider *et al.*, 2014). Rudolf *et al.* (1994) estimated the sampling error of monthly precipitation in 2.5^∘^ grid boxes over different land regions with high station coverage. Using 5 rain gauges per grid cell lead to sampling error between ±7 and 40% while using 10 stations reduces the error to ±5–20%.

While the interpolation of irregularly distributed rain‐gauge measurements onto regular grids is an additional error source, it also has several advantages. For example: 1) climate models can then be evaluated more directly since they represent spatial area averages rather than point data, 2) averaging over regions is straight forward, and 3) estimated data becomes available for non‐observed locations. However, interpolation tends to introduce excessive smoothing of spatial variability and may thus lead to an underestimation of extremes ( Haylock *et al.*, 2008; Hofstra *et al.*, 2010). Ly *et al.* (2011) investigated the influence of the gridding methods on the generation of a daily, high‐resolution gridded precipitation data set in a catchment in Belgium. They found root mean squared error (RMSE) differences of up to 10 mm day^− 1^ when only few raingages were used for the interpolation. When using more than one station per 300 km^2^, the RMSE differences are typically bellow 0.5 mm day^− 1^. Wagner *et al.* (2012) did a similar analysis for a data scarce catchment in India. They found that the gridding method lead to differences in the annual mean catchment precipitation of up to 50%. Furthermore, they showed the successful integration and large potentials of using spatial pattern from satellite derived precipitation products to generate gridded data sets in data scarce regions. Contractor *et al.* (2015) used different gridding methods to derive daily precipitation data sets over Australia and compared them with satellite derived products. They found that the data sets agree well for low to moderate daily precipitation amounts but start to diverge for values above 20 mm day^− 1^. Beguería *et al.* (2015) show that the spatial variance in gridded observational data sets depend on the spatial density of observations used for their construction. They conclude that this can lead to erroneous estimates of climate variability and extremes because most data sets have large temporal changes in the number of the underlying stations.

Inhomogeneities in precipitation records primarily impact analysis of climate change and trends. Effects of inhomogeneities at individual stations are in general reduced when the regional time series are analysed that include the average over multiple stations (New *et al.*, 1999). Hofstra *et al.* (2009), however, show that including a single inhomogeneous station may influence the homogeneity of a whole area in a gridded data set.

Precipitation is not only difficult to observe, it is also difficult to model because it encompasses processes that occur on a wide range of scales (from the micro to the synoptic‐scale). Many of these processes, such as deep convection or phase transitions, occur on sub‐grid scales and have to be parameterized in climate models. The parameters therein are partly used to tune the simulated precipitation with respect to the observations (Rotstayn, 2000; Räisänen, 2007; Bellprat *et al.*, 2012).

Even though it is widely known that observational data sets contain errors (e.g. Klein Tank *et al.*, 2002; Frei *et al.*, 2003; Hofstra *et al.*, 2009; Rauthe *et al.*, 2013; Isotta *et al.*, 2014, 2015; Schneider *et al.*, 2014). it is common practice to evaluate, statistically downscale, and bias correct climate model output with single observational data sets without addressing uncertainties (e. g., Jacob *et al.*, 2007; Kotlarski *et al.*, 2014; Prein *et al.*, 2013a; Dosio and Paruolo, 2011; Hirschi *et al.*, 2011). Herold *et al.* (2015) show that comparing measurements of the global average daily precipitation intensity over land from rain gauges, remote sensing, and/or reanalyses data sets leads to a similar spread than found in the Coupled Model Intercomparison Project Phase 5 (CMIP5) global climate model simulations. On a European scale, there are several studies that attempted to investigate uncertainties in gridded precipitation data sets. Klein Tank *et al.* (2002) and Hofstra *et al.* (2009) found that the continental‐scale European Observation (E‐OBS) data set shows inhomogeneities in time series and RMSE differences of up to 5.8 mm day^− 1^ with respect to regional data sets (RDs), exhibiting higher station densities. Isotta *et al.* (2015) compared precipitation from several European‐scale regional reanalyses (including the Hirlam Mesan Reanalysis (HMR) (Dahlgren *et al.*, 2014) and ERA‐Interim (Dee *et al.*, 2011); see Tables 1 and 2) with widely used observational data sets (E‐OBS, CRU (Harris *et al.*, 2014), GPCC (Schneider *et al.*, 2014), and EURO4M‐APGD which has a high station density in the Alps (Isotta *et al.*, 2014); see Tables 1 and 2). They show strengths (e.g. spatial variations, correction of unrealistic spatial features) and weaknesses (e.g., overestimate mean precipitation and wet day frequency) of the regional reanalyses. They state that low station density is a major error source and that observational data sets tend to agree in regions where their station densities are similarly high. Rauthe *et al.* (2013) compared the high‐resolution gridded daily data set HYRAS with precipitation from a station network in central Europe and found mean absolute errors of about 2 mm day^− 1^ and also highlight the need of a high station network. Highest differences to E‐OBS and PRISM (Parameter‐elevation Regression on Independent Slope Model; Schwarb (2000) and Schwarb *et al.* (2001)) were found in regions with complex orography. Kidd *et al.* (2012) compared several satellite‐based precipitation products over northwest Europe with ground stations. They found that the quality of the satellite products is lowest during winter and that they generally underestimate precipitation during all seasons.

**Table 1:** List of daily observational data sets.

Data set	Coverage and acronym	Period	Spacing and frequency	Average stations per 25 km × 25 km	Station ratio compared to E‐OBS
E‐OBS (v10.0) (Haylock *et al.*, 2008)	Europe	1950–2013	25 km daily	0.2–2	1
HMR (Dahlgren *et al.*, 2014)	Europe	1989–2010	5 km daily	0.1–4	2
EURO4M‐APGD (v1.2) (Isotta *et al.*, 2014)	European Alps (AL)	1971–2008	5 km daily	11	5.5
REGNIE (DWD, 2009)	Germany (GE)	1961–2015	1 km daily	5	2
aPTHBV (Johansson, 2002)	Sweden (SW)	1961–2010	4 km daily	2	0.8
aKLIMAGRID (Mohr, 2009)	Norway (NO)	1957–2013	1 km daily	3	5
Spain011 (Herrera *et al.*, 2012)	Spain (SP)	1971–2011	12 km daily	3.4	27
CARPATCLIM (Spinoni *et al.*, 2015)	Carpathians (CA)	1961–2010	10 km daily	0.8	5
UKCP09 (Perry and Hollis, 2005)	United Kingdom (UK)	1910–2011	5 km daily	11.3	33
bSAFRAN (Quintana‐Seguí *et al.*, 2008; Vidal *et al.*, 2010)	France (FR)	1958–2013	8 km hourly	4.5	44

a Corrected for observation losses.

b Regional reanalysis.

**Table 2:** List of monthly, low‐resolution observational data sets.

Data set	Coverage	Time period	Spacing and frequency	Input data
U‐DEL (Legates and Willmott, 1990)	Global land	1900–2010	0.5^∘^ monthly	∼24 600 land stations from GHCN v2 and a few other sources
CRU (Harris *et al.*, 2014)	Global land	1901–2012	0.5^∘^ monthly	∼4000 station records primarily from CLIMAT, Monthly Climatic Data from the World, and World Weather Records
GPCC (Schneider *et al.*, 2014)	Global land	1900–2013	0.5^∘^ Monthly	∼67 200 rain‐gauge stations
aGPCP (Adler *et al.*, 2003)	Global	1979–2015	2.5^∘^ monthly	6500–7000 rain‐gauge stations, satellites, and sounding observations
PREC (Chen *et al.* (2002)	Global land	1948–2015	0.5^∘^ monthly	∼17 000 GHCN v2 gauge measurements
ERA‐Interim (Dee *et al.*, 2011)	Global	1979–2015	∼79 km 3 hourly	Most *in situ* and satellite data used in numerical weather forecasting, including satellite radiances
aPERSIANN‐CDR (Ashouri *et al.*, 2015)	60^∘^S–60^∘^N	1983–2015	0.25^∘^ daily	Precipitation estimates derived from satellite infrared and microwave measurements are bias corrected with the GPCP monthly precipitation data set.

a corrected for observation losses.

**Figure 1:**
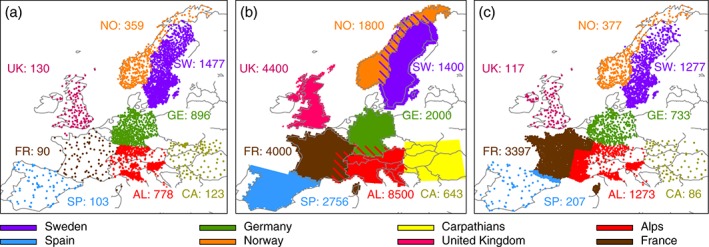
Stations contained in different regions of E‐OBS (panel a), the RDs, (panel b), and the HMR (panel c) data set and areal coverage of the eight RDs (panel b). The numbers in the panels give the approximate number of stations used to create the gridded data set in different regions. Only stations that cover more than 80% of the time period (1989–2008) are considered.

This study complements previous studies by expanding the investigations of uncertainties in gridded observational data sets to most land areas in Europe (eight subregions, see Figure 1(b)) within the period 1989–2008. This became recently possible, because many regional high‐resolution precipitation data sets (see Table 1 except for E‐OBS and Figure 1(b)) have become available in the last few years. We also include data sets with precipitation undercatch correction which allows us to estimate the uncertainty contribution from this frequently neglected error source. Additionally, we include frequently used European and global data sets to the analysis to provide a holistic overview of the uncertainties in precipitation estimates. Most importantly, we compare the derived observational uncertainties with errors in simulated precipitation from state‐of‐the‐art high resolution (0.11^∘^ horizontal grid spacing) regional climate model (RCM) simulations. This is hoped to provide a guideline for future studies focusing on model evaluation, model selection, model development, empirical‐statistical bias correction, and statistical downscaling of model results.

This study is structured as follows. In Section 2, we introduce the investigated observational and model data sets. In Section 3, we focus on daily precipitation from three sets of observational data sets and investigate the sources of differences in their precipitation estimates. In Section 4, we analyse monthly precipitation and additionally include seven global precipitation data sets that are based on surface stations, satellite observations, and reanalysis models. Section 5 shows the comparison between observational uncertainties and errors in modelled precipitation and highlights the importance of a multi observational data set approach for the evaluation of climate models. In Section 6, the results are discussed and Section 7 closes with conclusions.

## 2 Observational data sets and RCMs

We collected an ensemble of RDs, which provide high‐resolution (≤12‐km grid spacing) daily precipitation observations for eight regions in Europe (see Table 1). For the daily precipitation analysis, we additionally include the E‐OBS version 10 (Haylock et al., 2008) data set (25‐km grid spacing) and the recently established HMR (Dahlgren et al., 2014) regional reanalysis (5‐km grid spacing). Figure 1(b) shows the approximate number of stations that are included in each of the RDs and compares them to the number of stations that are included in the same region in E‐OBS (Figure 1(a)) and the HMR (Figure 1(c)). The station density in the RDs and the other two data sets is similar in Sweden. The RDs and the HMR have a similar station density in France while E‐OBS has approximately 40 times less stations. In the UK, Spain, Germany, and the Carpathians the station densities in the RDs are between 2 and 27 times higher than in the other two data sets (see Figure 1 and Table 1 right column).

The HMR and the RD of France (SAFRAN) differ from the other RDs and the E‐OBS because they are downscaled regional reanalyses. This means, the HMR and SAFRAN includes additional information from dynamical high‐resolution limited area models (Sass et al., 2002; Quintana‐Seguí et al., 2008; Vidal et al., 2010; Dahlgren et al., 2014). The HIRLAM reanalysis is downscaling ERA‐Interim data to a 22 km grid. Surface data are assimilated from ERA‐Interim and atmospheric data are assimilated from the ECMWF archives. In a second step, the MESAN analysis system is applied to downscale the HIRLAM reanalysis to a 5 km grid. For the downscaling of precipitation, data from the Swedish Meteorological and Hydrological Institute (SMHI), the Météo‐France, and the ECA&D database (Klein Tank et al., 2002) are used. In contrast, E‐OBS only includes data from the ECA&D database.

In addition to the daily E‐OBS, HMR, and RDs data sets, seven more coarsely resolved global observational data sets are included (see Table 2). The University of Delaware (U‐DEL; Legates and Willmott (1990)), Climatic Research Unit (CRU; Harris et al. (2014)); Global Precipitation Climatology Centre (GPCC; Schneider et al. (2014)), and Precipitation Reconstruction Land (PREC; Chen et al. (2002)) data sets are based on different sets of station observations and provide monthly precipitation on a 0.5^∘^ grid for all land areas of the world. In the Global Precipitation Climatology Project (GPCP; Adler et al. (2003)) data set, stations, satellites, and sounding observations are combined for monthly precipitation estimations on a 2.5^∘^ grid. The Precipitation Estimation from Remote Sensing Information using Artificial Neural Network – Climate Data Record (PERSIANN‐CDR; Ashouri et al. (2015)) is based on satellite observations that are bias corrected with the GPCP precipitation. PERSIANN‐CDR provides daily precipitation estimates for the region 60^∘^S–60^∘^N on a 0.25^∘^ grid. We did not include PERSIANN‐CDR in the daily analysis because it does only include the southernmost part of Sweden and Norway and it has large differences to the other observational data sets (except GPCP). Finally, the European Centre for Medium‐Range Weather Forecasts Interim reanalysis (ERA‐Interim; Dee et al. (2011)) precipitation is also included.

Several of the observational data sets are corrected for precipitation undercatch. The applied correction methods, however, are different. In the Norwegian RDs the method of Førland and Institutt (1996) is applied in which an exposure class is assigned to every station (see Mohr (2009)). For solid precipitation (e.g. snow) and extremely sheltered stations, the correction factor is 1.05 while for extremely exposed stations the correction factor is 1.8 (i.e. adding 80% more precipitation to the measured value). For rain, the correction factors are between 1.02 and 1.14. These values are derived for the Nordic gauges with wind shields. In the Swedish data set, all station are classified according to wind speed. The applied correction factors are lower than those used in Norway and vary between 1.015/1.04 and 1.12/1.36 for liquid/solid precipitation.

Undercatch correction is more challenging for global data sets because important information such as error characteristics of the gauges, their exposure, or the phase of precipitation are often not available (Schneider et al., 2014). Frequently, bulk correction factors for monthly climatological conditions are applied to global data sets such as GPCP and respectively PERSIAN‐CDR (Legates, 1987; Sevruk, 1989; Legates and Willmott, 1990). Those factors are provided on a climatological mean basis for each calendar month (Legates and Willmott, 1990) and vary between 1.0 and 3.0. (Legates and Willmott, 1990) and vary between 1.0 and 3.0. Fuchs et al. (2001) developed an improved correction method that takes the weather conditions (wind, temperature, relative humidity, precipitation phase, and intensity) into account. Schneider et al. (2014) state that this refined correction method leads to correction factors that are approximately 15% smaller than the bulk correction factors from factors from Legates and Willmott (1990). For the evaluation of modelled precipitation, the output from eight high resolution (0.11^∘^, approximately 12.5‐km horizontal grid spacing) RCM simulations from the European branch of the Coordinated Regional Climate Downscaling Experiment (EURO‐CORDEX) (Jacob et al., 2013) are used. The RCMs are forced by the ERA‐Interim reanalysis (Dee et al., 2011) on their lateral boundaries, and cover the period 1989–2008. A description of the included models and their basic setup can be found in Table 3.

**Table 3:** List of models.

Model; Institute	Physics	Soil spin‐up, land use, and vertical levels
ARPEGE (Déqué, 2010); Métó‐France	**RS**: Morcrette (1990); **CS**: Bougeault (1985); **MS**: Ricard and Royer (1993) Ricard Royer; **LSS**: Douville et al. (2000) Douville, Planton, Royer, Stephenson, Tyteca, Kergoat, Lafont, Betts; **BLS**: Ricard and Royer (1993) Ricard Royer	**SI**: Year 1989 is run twice; **VL**: 31
CCLM Böhm et al. (2006) Bohm, Kucken, Ahrens, Block, Hauffe, Keuler, Rockel, Will, Rockel et al. (2008) Rockel, Will, Hense; BTU	**RS**: Ritter and Geleyn (1992) Ritter Geleyn; **CS**:Tiedtke (1989); **MS**: Doms et al. (2011) Doms, Forstner, Heise, Herzog, Mironov, Raschendorfer, Reinhardt, Ritter, Schrodin, Schulz, Vogel, Baldauf and Schulz (2004) Baldauf Schulz; **LSS**: TERRA‐ML Doms et al. (2011) Doms, Forstner, Heise, Herzog, Mironov, Raschendorfer, Reinhardt, Ritter, Schrodin, Schulz, Vogel; **BLS**: Louis (1979)	**SI**: Initialization with climatological soil moisture; **LU**: GLC2000 Joint Research Centre (2003); **VL**: 40
HIRHAM Christensen et al. (1998) Christensen, Christensen, Machenhauer, Botzet; DMI	**RS**: Morcrette et al. (1986) Morcrette, Smith, Fouquart, Giorgetta and Wild (1995) Giorgetta Wild; **CS**: Tiedtke (1989); **MS**: Lohmann and Roeckner (1996) Lohmann Roeckner; **LSS**: Hagemann (2002); **BLS**: Louis (1979)	**SI**: Initialization with climatological temperatures and full water reservoirs. One year spin‐up. ; **LU**: USGS Hagemann (2002); **VL**: 31
RACMO van Meijgaard et al. (2012) van Meijgaard, Van Ulft, Lenderink, de Roode, Wipfler, Boers, Timmermans; KNMI	**RS**: Fouquart and Bonnel (1980) Fouquart Bonnel, Mlawer et al. (1997) Mlawer, Taubman, Brown, Iacono, Clough; **CS**: Tiedtke (1989); Nordeng (1994); Neggers et al. (2009) Neggers, Koehler, Beljaars; **MS**: Tiedtke (1993); Tompkins et al. (2007) Tompkins, Gierens, Radel, Neggers (2009); **LSS**: Van den Hurk et al. (2000) Van den Hurk, Viterbo, Beljaars, Betts, Balsamo et al. (2009) Balsamo, Viterbo, Beljaars, van den Hurk, Hirschi, Betts, Scipal; **BLS**: Lenderink and Holtslag (2004) Lenderink Holtslag, Siebesma et al. (2007) Siebesma, Soares, Teixeira	**SI**: Initialized from ERA‐Interim on 1979.01.01 00:00; **LU**: ECOCLIMAP (1 km) Champeaux et al. (2003) Champeaux, Masson, Chauvin, Masson et al. (2003) Masson, Champeaux, Chauvin, Màiguet, Lacaze; **VL**: 40
RCA Samuelsson et al. (2011) Samuelsson, Jones, Willà, Ullerstig, Gollvik, Hansson, Jansson, Kjellstrom, Nikulin, Wyser; SMHI	**RS**: Savijarvi (1990); Sass et al. (1994) Sass, Rontu, Savijarvi, Raisanen; **CS**: Kain and Fritsch (1990) Kain Fritsch, Kain and Fritsch (1993) Kain Fritsch; **MS**: Rasch and Kristjánsson (1998) Rasch Kristj sson; **LSS**: Samuelsson et al. (2011) Samuelsson, Jones, , Willà, Ullerstig, Gollvik, Hansson, Jansson, Kjellstrom, Nikulin, , Wyser; **BLS**: Cuxart et al. (2000) Cuxart, Bougeault, Redelsperger	**SI**: Initialized from ERA‐Interim on 1979.01.01 00:00; **LU**: ECOCLIMAP (1 km) Champeaux et al. (2003) Champeaux, Masson, Chauvin, Masson et al. (2003) Masson, Champeaux, Chauvin, Màiguet, Lacaze; **VL**: 40
REMO Jacob et al. (2012) Jacob, Elizalde, Haensler, Hagemann, Kumar, Podzun, Rechid, Remedio, Saeed, Sieck, Teichmann, Wilhelm; CSC	**RS**: Morcrette et al. (1986) Morcrette, Smith, Fouquart; **CS**:Tiedtke (1989); Nordeng (1994); Pfeifer (2006); **MS**: Lohmann and Roeckner (1996) Lohmann Roeckner; **LSS**: Hagemann (2002); Rechid et al. (2009) Rechid, Hagemann, Jacob; **BLS**: Louis (1979)	**SI**: Soil initialized from ERA‐Interim. No Spin‐up; **LU**: USGS Hagemann (2002); **VL**: 27
WRF 1 Skamarock et al. (2008) Skamarock, Klemp, Dudhia, Gill, Barker, Wang, Powers; CRP‐GL	**RS**: CAM 3.0 Collins et al. (2004) Collins, Rasch, Boville, McCaa, Williamson, Kiehl, Briegleb, Bitz, Lin, Zhang, Dai; **CS**: Modified Kain (2004); **MS**: WSM 6‐class Hong and Lim (2006) Hong Lim; **LSS**: NOAH Ek et al. (2003) Ek, Mitchell, Lin, Rogers, Grunmann, Koren, Gayno, Tarpley; **BLS**: YSU Hong et al. (2006) Hong, Noh, Dudhia	**SI**: Soil initialized from ERA‐Interim. No Spin‐up; **LU**: IGBP‐MODIS (30′′); **VL**: 50
WRF 2 Skamarock et al. (2008) Skamarock, Klemp, Dudhia, Gill, Barker, Wang, Powers; IPSL and INERIS	**RS**: RRTMG Lacono et al. (2008) Lacono, Delamere, Mlawer, Shephard, Clough, Collins; **CS**: Grell and Devenyi (2002) Grell Devenyi; MS: Hong et al. (2004) Hong, Dudhia, Chen; **LSS**: NOAH Ek et al. (2003) Ek, Mitchell, Lin, Rogers, Grunmann, Koren, Gayno, Tarpley; **BLS**: YSU Hong et al. (2006) Hong, Noh, Dudhia	**SI**: Soil initialized from ERA‐Interim. No Spin‐up; **LU**: USGS Land Use; **VL**: 32

**BLS** = boundary layer scheme; **CS** = convection scheme; **LSS** = land‐surface scheme; **LU** = land use; **MS** = microphysics scheme; **RS** = radiation scheme; **SI** = soil initialization; **VL** = vertical levels.

All analyses are performed on common grids. The daily precipitation analyses in Section 3 are performed on the 0.25^∘^ regular grid of E‐OBS while the large scale, monthly and model analyses are done on a 0.5^∘^ regular grid. A conservative remapping routine is used, which takes weighted averages of the precipitation on the source grid to calculate precipitation on the target grid (e.g. Jones, 1999). The weights are proportional to the area of a target grid cell covered by a source grid cell. The benefit of using a conservative remapping method compared to, e.g. bilinear interpolation, is that the former conserves the integral of precipitation over any domain. We decided to upscale the high‐resolution data sets to coarser grids to not penalize the coarser resolved data sets due to missing small‐scale features. Furthermore, the observational data sets and the model simulations typically have an effective resolution (actual information content) of more than four times their grid spacing (Skamarock, 2004; Prein et al., 2013b; Isotta et al., 2015).

## 3 Comparison of daily precipitation data sets

In this Section, we investigate the differences between the daily precipitation from the RDs, the HMR, and E‐OBS data set and assign them to different error sources.

**Figure 2:**
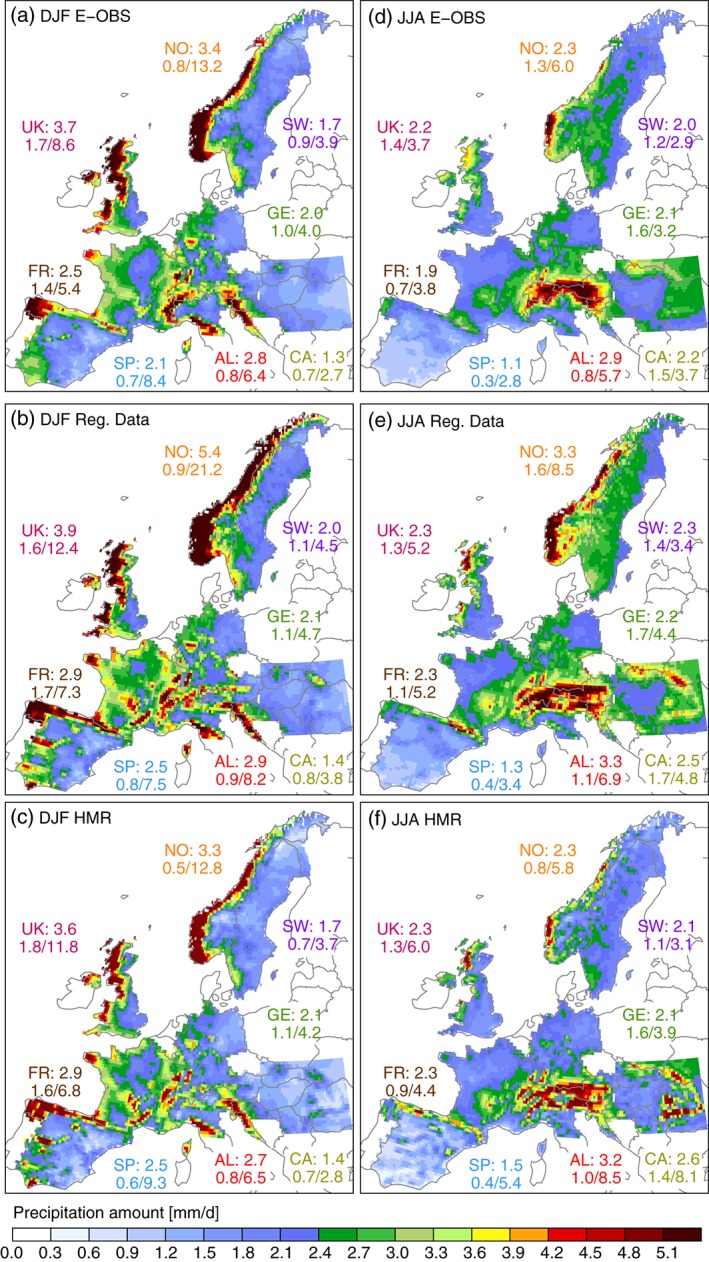
Seasonal average precipitation in DJF (left) and JJA (right) for E‐OBS (first row), the RDs (second row), and the HMR (third row). Shown beside the regions is the mean, minimum, and maximum seasonal average precipitation of the grid cell values in the region. The Alpine data set applies to the red hatched areas in Figure 1(b)) while in Norway results from the Norwegian data set are shown.

Average winter (December, January, February; DJF) precipitation is highest in mountainous regions (Figure 2(a)–(c)). During summer (June, July, and August; JJA; Figure 2(d)–(f)), the Mediterranean region is drier than in DJF while the European Alpine area and the Carpathians are wetter due to the frequent occurrence of thunderstorms.

While these larger scale patterns are similar in all data sets, regional patterns can be very different. For example, there is a well‐observed precipitation minimum in the inner Alpine region during JJA (e.g. Cebon *et al.*, 1998)which is captured by the RDs and the HMR but missed in the E‐OBS data set (compare Figure 2(d) with Figure 2(e) and (f)) (*cf* Isotta *et al.*, 2015). Other examples are the JJA precipitation in the Carpathians (spatially homogeneous in E‐OBS, distinct peaks over the mountains in the RDs and the HMR) or the Eastward extend of high precipitation amounts on the Atlantic Coast and the Pyrenees in Spain during DJF.

**Figure 3:**
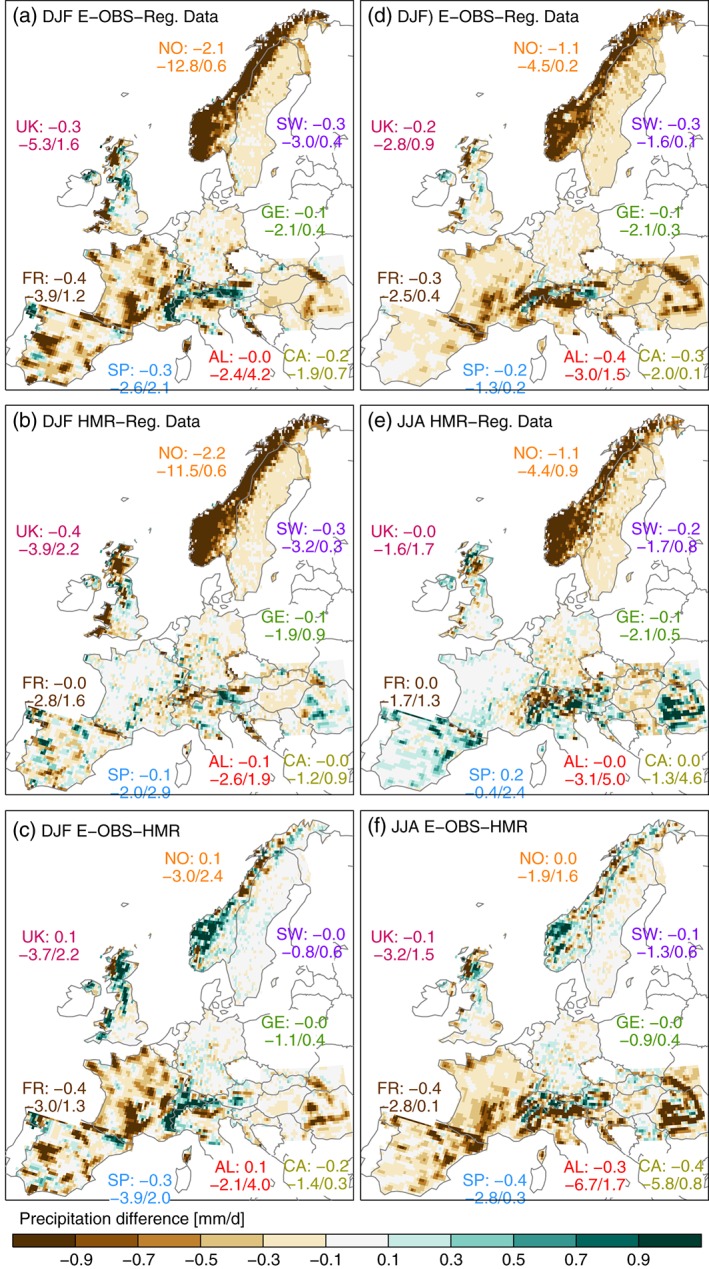
Same as in Figure 2 but for differences between E‐OBS and the RDs (first row), HMR and E‐OBS (second row), and E‐OBS and HMR (third row). The numbers in the panels are as in Figure 2 but for precipitation difference.

The observational data sets do not only disagree in the regional spatial patterns, but also in the amount of seasonal average precipitation (Figure 3). Most striking are the differences in Norway. While E‐OBS is on average similar to HMR, the RD shows ∼2 mm day^− 1^ (∼60%) more precipitation during DJF and ∼1 mm day^− 1^ (∼40%) more during JJA. Grid cell differences reach up to ∼13 mm day^− 1^ (∼80%). Averaged over the investigated regions, the RDs feature more precipitation than the HMR and E‐OBS, even though there are small areas where they are drier (e.g. parts of the Iberian Peninsula, the inner Alpine region, or the East coast of the UK). Remarkable is the difference between the RDs and the HMR during JJA along the French boarders to Italy, Switzerland, and Spain (panel e). There are only minor differences between the two data sets in France but differences are getting large across the boarders. This is not dependent on orography since the Pyrenees and the Alps do well extend into France, but is rather related to changes in the station density of the HMR (see Figure 1(c)), which is supported by findings of Isotta *et al.* (2015) for the Alpine area. This result demonstrates the importance of a high station density network for the accurate estimation of precipitation. Furthermore, it indicates that missing information in regions with low station densities cannot easily be provided by state‐of‐the‐art reanalyses with dynamical models, such as attempted in the HMR.

The reasons for the large differences between the observational data sets may be manifold, but two major aspects can be easily identified. The first one is gauge undercatch correction. Norway and Sweden exhibit a relatively high station density in all data sets, thus the station density is not the primary reason for differences. However, the RDs are corrected for gauge undercatch in these regions, which is probably the dominant source for differences. In the Norwegian data set, correction factors of up to 14%/80% for liquid/solid precipitation (Mohr, 2009) are applied to extremely unshielded locations (Førland and Institutt, 1996). This explains the maximum differences of 80% during DJF in Norway since in this season precipitation falls predominantly as snow. In Sweden, the differences are smaller because of less exposure of the rain gauges to wind (smaller correction factors) and the lower precipitation amounts falling in DJF (see Figure 2). In the other RDs, in E‐OBS, and in the HMR, no undercatch correction is applied, implying that other aspects must be responsible for further differences.

**Figure 4:**
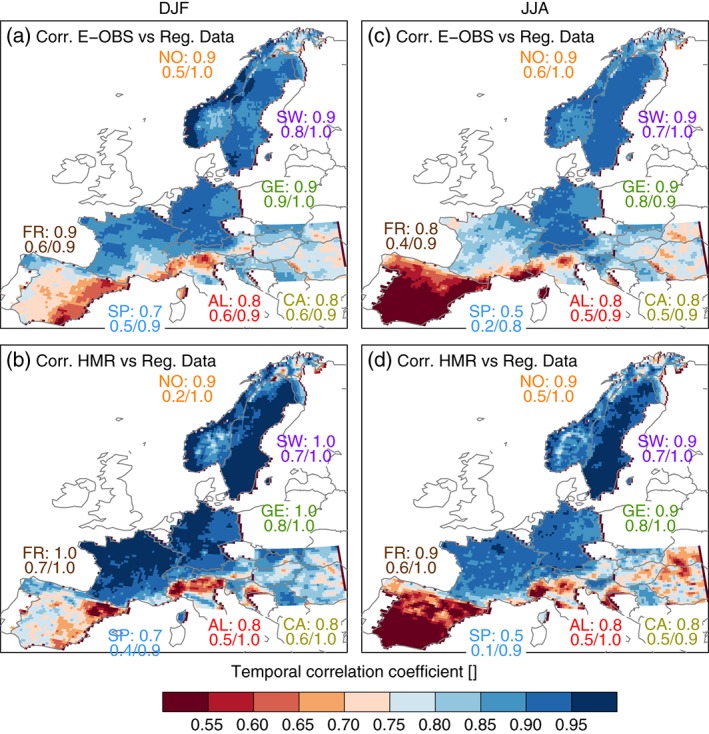
Temporal Spearman rank correlation coefficients of daily grid cell precipitation between E‐OBS and the RDs (first row) and HMR and RDs (second row) for DJF (left) and JJA (right). UK is not shown because the RD contains only monthly data. The numbers in the panels are as in Figure 2 but for correlation coefficients.

Station density (sampling errors) appears to be the second major source of differences between the data sets. Figure 4 depicts the Spearman rank correlation coefficient for daily time series on grid‐cell basis comparing E‐OBS and the RDs (panels a and c) and the HMR and the RDs (panels b and d) in DJF and JJA, respectively. We assume that in grid cells where a data set has no stations, it is likely that precipitation events observed in other data sets are missed or underestimated. This leads to a reduced temporal correlation. Temporal correlation is barely affected by undercatch correction, since it is rather insensitive to the scaling of the time series. Figure 4 reveals low correlation coefficients for areas where at least one of the compared data sets has a low station density meaning that daily precipitation time series at these locations differ strongly.

In general, correlation coefficients are higher in DJF than in JJA due to the predominant convective character of precipitation and its therefore high spatio‐temporal variability in JJA. This means that a high station density is even more important in JJA than in DJF. In the Alpine region, for example, E‐OBS has on average 2.0 stations per grid cell. However, the density is varying in different countries. Therefore, in Austria (0.1 stations per grid cell) we find low correlation coefficients, whereas in Slovenia (6.5 stations) high correlation coefficients are present. For the same reason, a similarly strong spatial gradient is shown between very high correlation coefficients in France and low ones in Spain and North‐Western Italy when the HMR is compared to the RDs in JJA (panel d). The correlation coefficients between the HMR and the E‐OBS data (not shown) are generally higher than the coefficients from the comparison to the RDs in all regions except France because these data sets use a similar station basis (except in France and Sweden; in E‐OBS, the station density is also high in Sweden).

**Figure 5:**
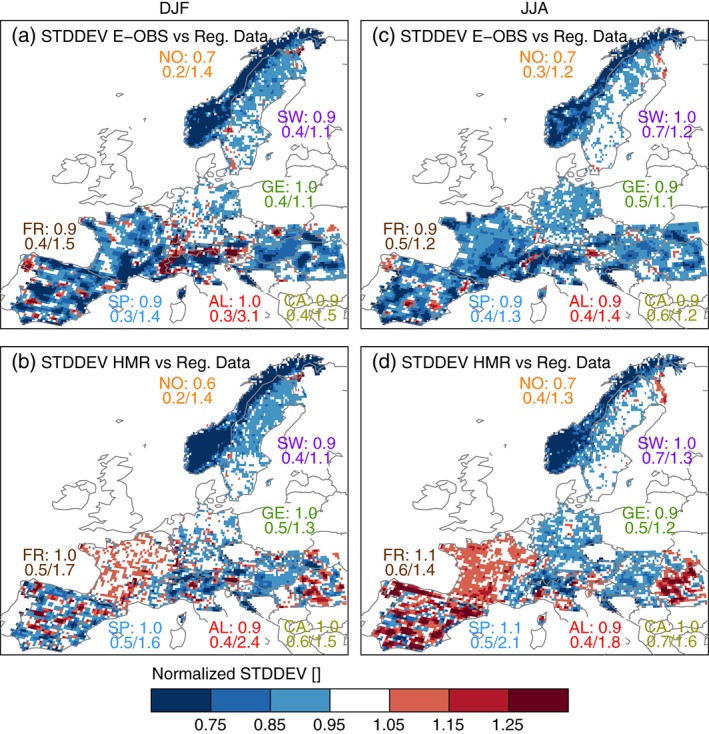
Standard deviations of daily grid cell precipitation of the E‐OBS/HMR data set (first/second row) divided by the standard deviations of the RDs for DJF (left) and JJA (right). The numbers in the panels are as in Figure 2 but for standard deviations.

Similar to the temporal correlation coefficients, also the daily variability, measured as temporal standard deviation (Figure 5), shows highest differences in regions where the station density varies between the different data sets. Additionally, undercatch correction tends to increase the temporal variability because of the amplification of precipitation events. Except for some small‐scale areas, E‐OBS has lower temporal variability than the regional data sets. The only exception is a continuous strip of higher variability on the southern side of the Alpine ridge during DJF. The regions with the most similar variabilities are Germany and Sweden during JJA. Temporal variabilities are more similar between the HMR and the RDs during DJF (especially in France where both have a high station density). Remarkable is the increased variability in the HMR compared to E‐OBS in Spain during JJA because the same set of stations (except in the Pyrenees) is used in both data sets. The additional information from the HIRLAM reanalysis might be responsible for the differences between E‐OBS and the HMR. A similar feature can also be seen in the Carpathians.

**Figure 6:**
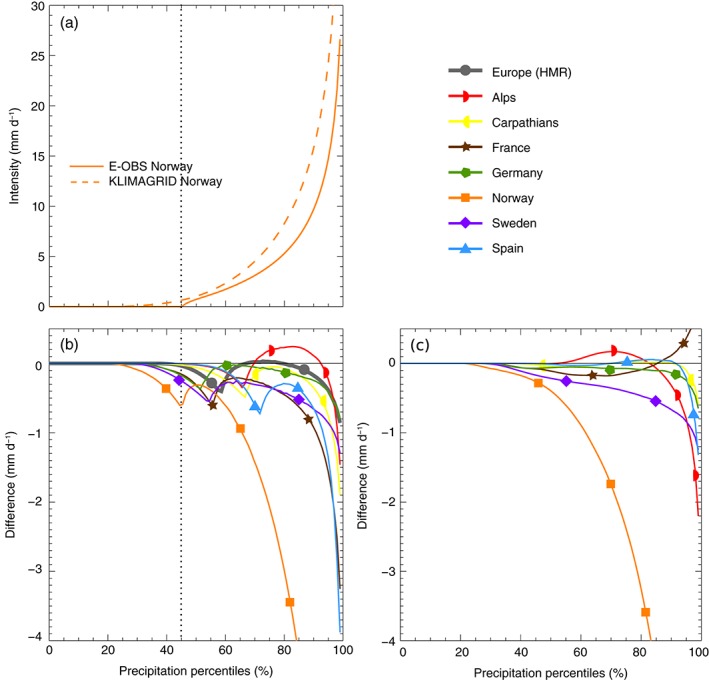
Empirical quantile functions of daily precipitation from E‐OBS and the KLIMAGRID data set in Norway during DJF (panel a). Differences between the quantile functions (E‐OBS minus RDs) in all regions during DJF are shown in panel b. The grey dotted line depicts the percentile below which E‐OBS has zero precipitation in Norway. Differences between the quantile functions of the HMR and the RDs are shown in panel c.

A further source of uncertainties in gridded observational data sets arises by interpolating point measurements to a grid. Thereby, extreme precipitation is affected most (Haylock *et al.*, 2008) This is a well‐known problem in E‐OBS (Haylock *et al.*, 2008; Hofstra *et al.*, 2009, 2010). Figure 6(a) depicts the daily empirical quantile functions for Norway during DJF. The difference between the two lines (E‐OBS minus KLIMAGRID) is shown in Figure 6(b) together with differences observed in the other RDs and the HMRs. All RDs and the HMRs have higher extreme precipitation values than E‐OBS. The lower extreme precipitation intensities in E‐OBS are likely caused by its coarser grid spacing (25 km) compared to the RDs and the HMRs (≤12 and 5 km, respectively). Typically the effective resolution of gridded observational data sets is several times larger than their grid spacing. For example, Isotta *et al.* (2014) estimated that the effective resolution of the EURO4M‐APGD data set is between 10 and 25 km (typical station spacing) compared to its 5‐km grid spacing. On larger scales (coarser grids), the evaluation results might therefore be more similar. The comparable grid spacing is probably also one reason why extremes are more similar in the HMR and RDs. The HIRLAM reanalysis appears to add information to the HMR as E‐OBS strongly underestimates extreme precipitation in Spain which is corrected in the HMR even though both data sets have the same station basis (compare blue line in Figure 6(b) with (c)). Highest differences are shown for Norway where the RD has much higher precipitation values because of the applied undercatch correction.

Certain technical specifications employed in the generation of gridded data sets also introduce uncertainties. For example, in E‐OBS, the threshold for rain days is defined as more than 0.5 mm day^− 1^ observed at a station. Therefore, the gridded data set only contains very few precipitation values between 0  and 0.5 mm day^− 1^. Instead, E‐OBS typically has 50% more dry days than the RDs or the HMR, which employ no such threshold. This gives rise to negative differences for low percentiles. The percentiles up to which E‐OBS has zero precipitation can be seen at the location of the local minimum in the lines in Figure 6(b)) (e.g. grey‐dotted line at 45% for Norway). Comparing the RDs to the HMR (Figure 6(c)) does not show such a feature.

**Figure 7:**
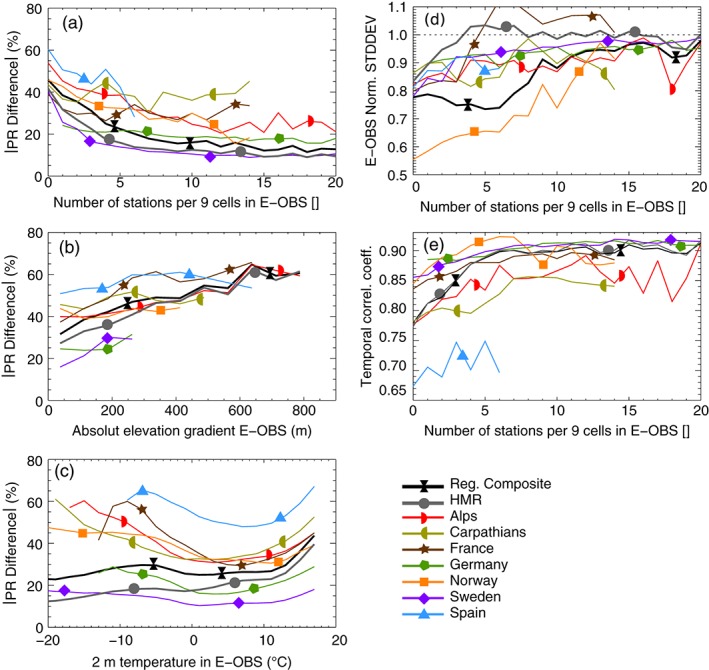
Median absolute differences in daily precipitation are shown dependent on the E‐OBS station density in the eight adjacent grid cells around a cell (panel a), the mean absolute elevation gradient to the adjacent grid cells (panel b), and the 2 m temperature in E‐OBS (panel c). Panel d shows the median (over grid cells) temporal standard deviation of daily precipitation from the data sets divided by the standard deviation of E‐OBS as a function of E‐OBS station density. Panel e shows the same for the Spearman rank correlation coefficients.

Facing these uncertainties, how large are the regional scale differences in the observational data sets? Figure 7(a) shows median absolute differences between the data sets (for precipitation ≥ 1 mm day^− 1^) dependent on the number of E‐OBS stations within an area of 3 × 3 grid cells. In case of zero stations, median differences are between 40 and 60% while they are between 10 and 30% for more than ∼9 stations (except for France and the Carpathians). The RDs composite has slightly higher differences to E‐OBS than the HMR but both data sets show a very similar relation to the station density. Differences are increasing for grid cells in complex orography (Figure 7(b)). There is a linear relationship between the absolute difference of precipitation at a grid cell and the absolute median elevation difference to its eight adjacent grid cells between ∼2.5% per 100 m in Germany, Norway, and the Carpathians, ∼5% in the Alps, Spain, and France, and ∼10% in Sweden. The RDs composite and the HMR have similar slopes of about 6% per 100 m. This is related to the high spatial heterogeneity of precipitation in complex terrain, the usually low station density in mountainous areas, and the higher fraction of solid precipitation. Furthermore, differences depend on 2 m temperature (taken from E‐OBS; Figure 7(c)) and are lowest between 0 and 12 ^∘^C and increase for higher (e.g. convective precipitation) and lower (e.g. snow undercatch) temperatures. The RDs composite and the HMR show the same kind of behavior with a steep increase in differences above 13 ^∘^C and a slight increase below zero. However, the differences tend to decrease again for temperatures below −5 ^∘^C and reach their minimum at −20 ^∘^C. The investigation of this unexpected behavior is beyond the scope of this study because the amount of data points in this temperature range is small and therefore barely influences the overall statistics.

The dependence of the temporal standard deviation of E‐OBS divided by the standard deviation of the RDs and the HMR is shown as a function of E‐OBS station density in (Figure 7(d)). In areas with no E‐OBS stations, the standard deviation is approximately 20% smaller (except for Norway where it is 45% smaller). This difference decreases with increasing station density but stays slightly negative, except for the RD of France and the HMR. Standard deviation differences stay constant (−25%) in the RDs composite for areas with less than six grid cells and quickly decreases afterwards. Finally, temporal Spearman rank correlation coefficients (Figure 7(e)) are more similar in regions with high station densities of E‐OBS (above 0.83 in regions with >9 stations) than in data sparse regions (0.77–0.86 in regions with no stations; except for Spain). The HMR has higher correlation coefficients than the RDs composite in regions with <15 stations.

It should be noted that the analysis in Figure 7 depends on the number of included grid points. Nine grid cells are reasonable because the spatial interdependence between two precipitation time series is strongly degrading with increasing distance Ly *et al.*, 2011; Prein *et al.*, 2013b).

## 4 Comparison of coarser resolution monthly precipitation

**Figure 8:**
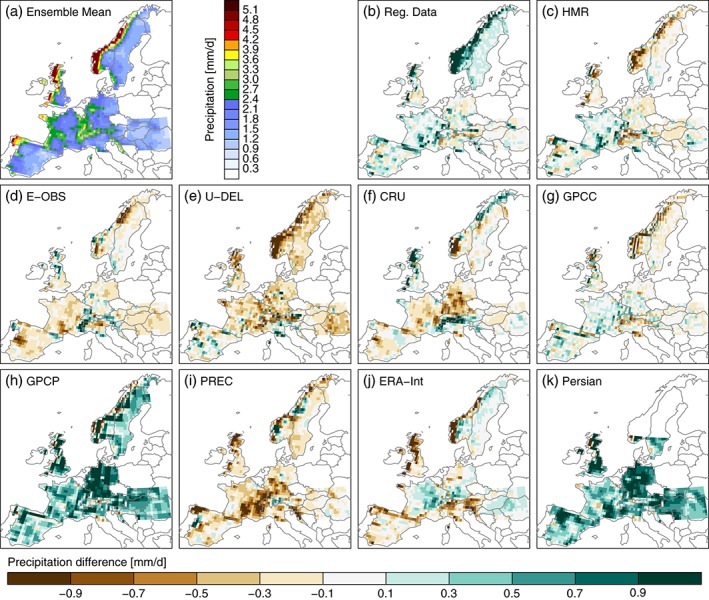
Mean DJF precipitation from the average of the observational data sets (excluding PERSIAN‐CDR) (panel a). The differences between the mean DJF precipitation in the individual observational data sets and the observational data sets average is shown in panels b–k.

**Figure 9:**
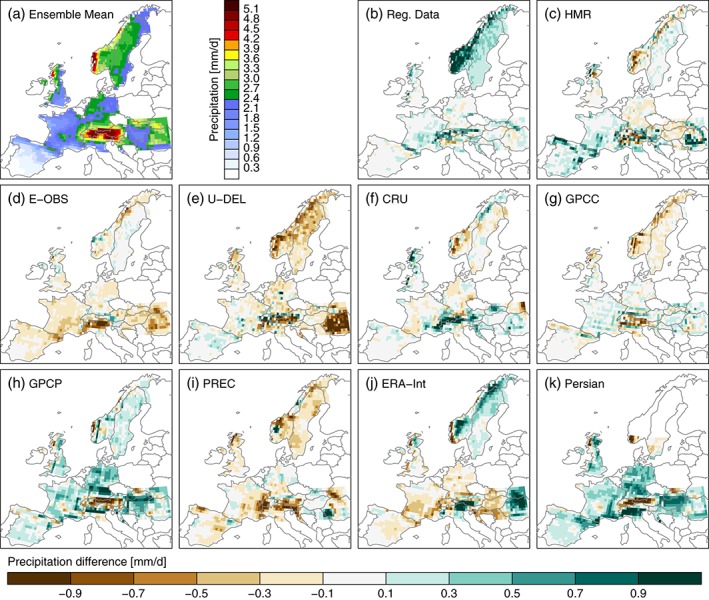
Same as Figure 8 but for JJA precipitation.

In addition to the RDs, the HMR, and the E‐OBS data set, we include seven more large‐scale precipitation data sets to our analysis in this Section (see Table 2 for an overview). All data sets are conservatively remapped to a regular 0.5^∘^ grid, which is larger or equal to the grid spacing of the data sets (except for the GPCP and the ERA‐Interim reanalysis; see Tab. 2), and averaged to monthly mean values. The mean of all observational data sets (excluding PERSIANN‐CDR) is used as reference for the evaluations shown in Figures 8, 9, and 11. This is beneficial because we can directly see how the individual data sets are performing with respect to their average, outliers get easily visible, and we do not have to subjectively select a single reference data set.

Figure 8(a) shows the observation ensemble mean precipitation in DJF. Compared to the higher resolution evaluation, many details got lost because of the spatial upscaling and the averaging over multiple data sets (*cf* Figure 2). Compared to the ensemble mean, the RDs (panel b) are predominantly wetter, which is most pronounced in Scandinavia due to the undercatch correction. Largest differences appear in the GPCP data set (panel h), which shows much more precipitation than all the other data sets (except the RDs in Norway) probably because of the applied undercatch correction. Since GPCP is used for the bias correction of PERSIANN‐CDR (panel k), both data sets show very similar seasonal average precipitation. Predominantly drier than the ensemble mean are the U‐DEL and PREC precipitation values (panels e and i). The former is especially dry in Norway while the later is driest in Southeastern France and Southern Germany. Most similar to the ensemble mean is the GPCC precipitation (panel g). Also the ERA‐Interim reanalysis precipitation (panel j) is close to the mean except for the coast of Norway, Northern UK, and the Alps. It should be mentioned that ERA‐Interim has no undercatch issues because precipitation is a simulated variable in the reanalysis.

In JJA (Figure 9), the spread between the different observations is smaller than in DJF but the basic patterns in the differences between the individual data sets are similar. Again, the RDs (panel b) have predominantly more precipitation than the ensemble mean with largest differences in mountainous regions. Compared to DJF, the positive difference is less pronounced in the GPCP and PERSIAN‐CDR precipitation (panels h and k) but in JJA, they show distinctly less precipitation over the Alps. U‐DEL and PREC (panels e and i) show again below‐average precipitation and have largest differences in the Alps and the Carpathians. ERA‐Interim precipitation (panel j) shows distinct positive differences in the Alps, the Carpathians, and the Scandinavian Mountains.

## 5 Influences on climate model evaluation

In this section, we show how uncertainties in gridded observational precipitation data sets compare to biases in state‐of‐the‐art climate models and affect their evaluation.

**Figure 10:**
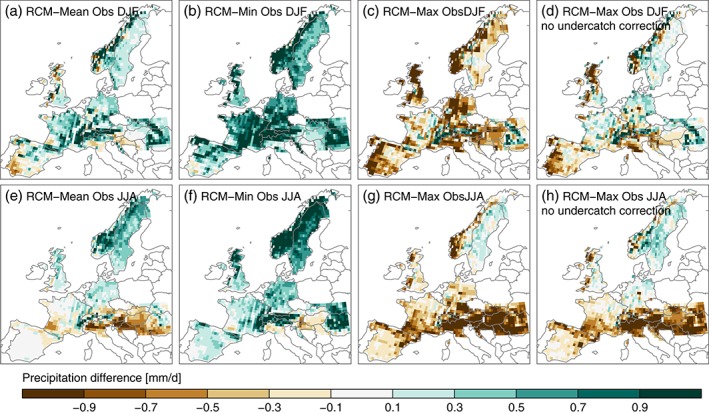
Differences between the multi‐model mean precipitation and the mean (panel a), minimum (panel b), maximum (panel c), and maximum without considering undercatch corrected data sets (panel d) precipitation from the observational data sets. Results for DJF/JJA average precipitation differences are shown in panels a–d/e–h respectively.

Comparing the multi‐model mean precipitation with the observational ensemble mean shows a predominant wet bias, which is most pronounced over mountain regions in DJF (Figure 10(a)). To understand if these biases are significant compared to the observational uncertainties, we investigate the differences between the multi‐model mean precipitation and the minimum/maximum precipitation in the observation ensemble (panel b/c). We consider a dry/wet bias as significant if the simulated precipitation is smaller/larger than the minimum/maximum precipitation in the observation ensemble. A significant dry bias only occurs in the Southern parts of Spain, while significant wet biases are found along the main Alpine Crest, the Carpathians, and the Pyrenees. Figure 10(d) shows the multi‐model mean minus the maximum precipitation excluding the observations with undercatch correction. In this case, significant wet biases are additionally found in Northern France, Northern Germany, Southeast England, and large parts of Scandinavia.

During JJA, mean modelled precipitation is smaller than the mean observed precipitation Southward of the Alps, the Southwest of France, and Southeast Europe (except the Carpathian Mountains, Figure 10(e)). More precipitation is modelled along all major mountain ranges, Germany, the Northern UK, and Scandinavia. Significant is the dry bias in Southeastern Europe, the Po Valley, and a small area North of the Pyrenees (panel f) while only the wet bias in Sweden and parts of Norway is significant (panel g). Excluding observations with undercatch corrections leads to additional significant wet biases in Northern Germany and Southeastern Norway (panel h).

The results in Figure 10 show that evaluating climate model precipitation with single observational data sets leads to a non‐representative bias pattern that can misguide further model development and the ranking of models in an ensemble. In large areas of Europe, the sign of the model bias is changing when evaluation is performed with different observational data sets. The observational uncertainty is largest in mountainous regions and especially in the Scandinavian Mountains due to accounting for precipitation undercatch in the RSs. In literature, it is frequently stated that state‐of‐the‐art RCMs are ‘too wet’ in large parts of Europe and particularly over mountainous regions, which is interpreted as a common RCM deficiency (Jacob et al., 2007; Kjellström et al., 2010; Kotlarski et al., 2014). However, our evaluation shows that most of these biases are not significant because they depend on the selected observational data set.

Two remarks are important to consider in the above evaluation. First, in this framework the significance of the model biases depends on the included observational data sets. It might be reasonable to exclude single data sets if they show unrealistic precipitation values or weight the observations, which is outside the scope of this study. Second, biases of single models are typically larger than the bias in the multi‐model mean because model biases tend to cancel out by averaging (Reichler and Kim, 2008). This topic will be addressed in the upcoming paragraphs.

**Figure 11:**
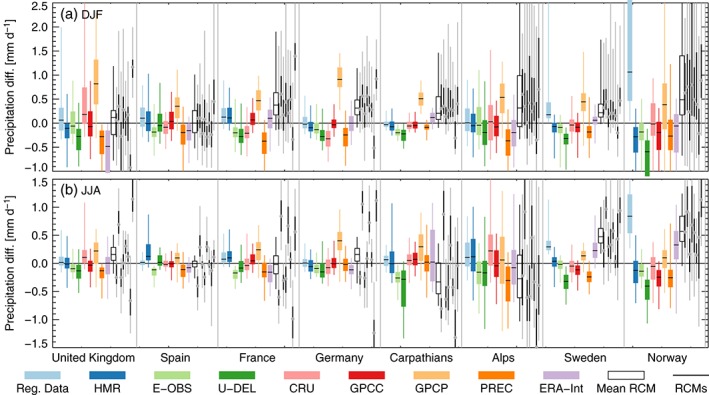
Box‐Whisker statistics showing the spatial spread for seasonal mean precipitation biases between the precipitation in the mean observational data set and the precipitation in the individual observations (colored boxes), the mean model (empty box), and the individual models (thin black boxes with circles showing the median). The boxes show the 25–75 quantile distance while the whiskers show the 5–95 quantile range. Results for DJF/JJA are shown in panel a/b.

Figure 11 shows an overview of seasonal mean model biases compared to observational uncertainties in a box‐whisker diagram. Observation mean precipitation is used as reference (zero line). The thick black lines (grey circles in case of the individual models) in the boxes show the median differences while the lower/upper box length show the 25/75 percentiles. The upper/lower whisker show the 5/95 percentile of the differences. As multi‐model mean biases and observational uncertainties are of similar size (empty box overlaps with the coloured boxes except for Sweden in JJA), quantitative and qualitative investigation of model biases clearly demands the consideration of observational uncertainties. Even though biases of single models spread more than the bias in the multi‐model mean, most biases of single models are still within the range of observational uncertainties (except for France, UK, and Germany during JJA; see grey vertical lines in Figure 11). In DJF, the individual models have more similar biases than in JJA, which is probably related to the convective nature of summertime precipitation and the high uncertainties in deep convection parameterization schemes applied in the models. There are two remarkable outliers in the observational ensemble. The first is the Norwegian RD due to its consideration of precipitation undercatch. The second is the GPCP data set (especially during DJF in Germany and the Carpathians). In GPCP, a bulk undercatch correction method is used that overestimates the undercatch by approximately 15% (Schneider et al., 2014). Furthermore, the GPCP data set has by far the largest grid spacing (2.5^∘^) of all considered data sets. The consequence is a spatially smooth precipitation field that underestimates precipitation maxima (e.g. in mountains) and overestimates precipitation in their surroundings (e.g. foothills) when compared to higher resolved data sets.

**Figure 12:**
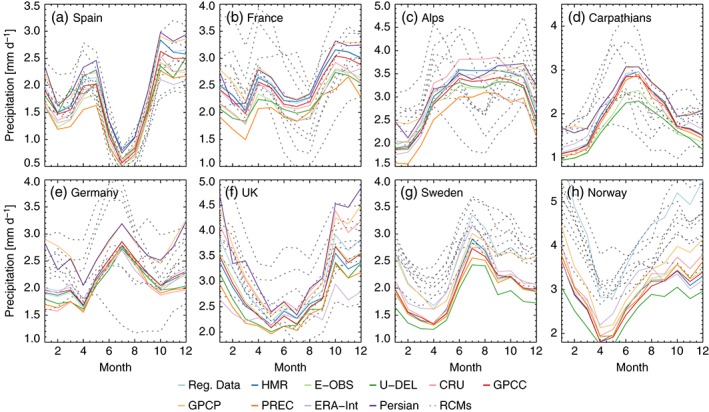
Annual cycle of monthly mean precipitation in different sub‐regions (panels). Results from the observational data sets are shown as coloured lines while precipitation from climate models is shown as grey‐dotted lines.

Beside the partly large observational uncertainties in seasonal mean precipitation, the observational data sets have a similar shape of the annual cycle of precipitation (coloured lines in Figure 12). The amplitude of the annual cycle can, however, differ largely. For example, in the UK (panel f) the ERA‐Interim annual cycle has a December maximum of 2.8 mm day^− 1^ and a May minimum of 2.1 mm day^− 1^, while the PERSIAN‐CDR annual circle has a December maximum of 4.9 mm day^− 1^ and a July minimum of 2.3 mm day^− 1^. In general the observational uncertainties tend to be larger during the winter season. We can again identify the same outliers as in Figure 11. The precipitation undercatch corrected Norwegian RD and the GPCP (and respectively the PERSIAN‐CDR) annual cycle have a positive offset from the other observations especially during DJF. The offset of GPCP/Persian‐CDR is highest in Germany.

Most climate models (grey‐dotted lines in Figure 12) are able to reproduce the shape of the observed annual cycle in Spain, the UK, Sweden, and Norway (panels a, f, g, and h), however, the shapes deviate largely in the other areas. For example, in the Alps (panel c), there is an observed summertime maximum in precipitation caused by the high convective activity in this seasons. The models, however, show spring and autumn maxima and a secondary summertime minimum. This is likely related to a misrepresentation of convective precipitation in the models due to error prone deep convection parameterizations Molinari and Dudek, 1992; Romps, 2010; Jones and Randall, 2011). In Sweden and Norway (panels g and h) modelled precipitation has a positive offset to most observations except for the undercatch corrected Norwegian RD.

**Figure 13:**
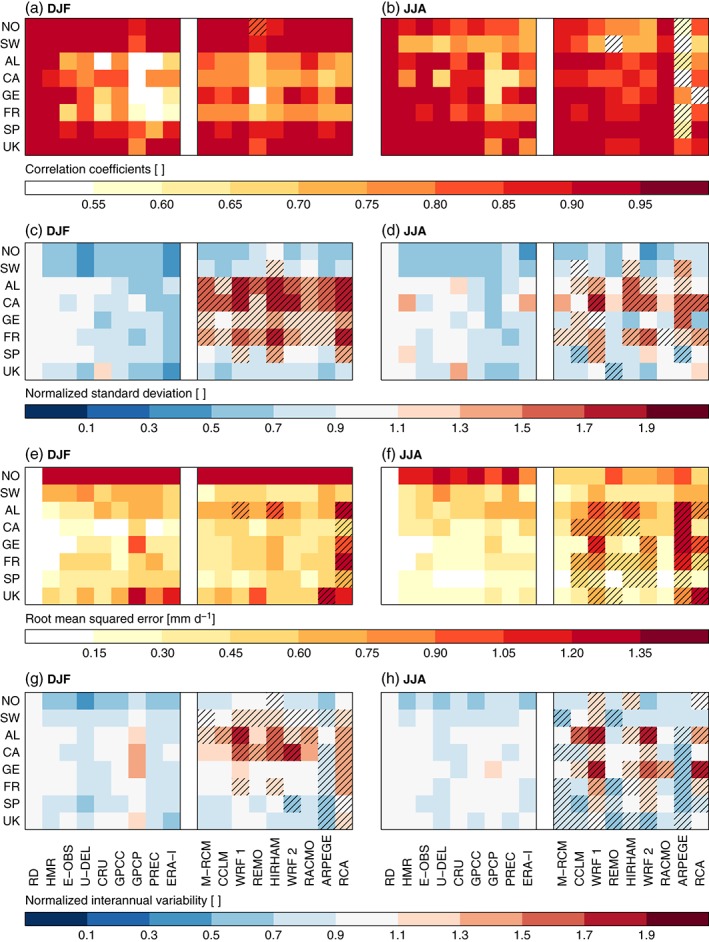
Heat maps showing spatial correlation coefficients (panels a and b), spatial standard deviations divided by the standard deviation of the RDs (panels c and d), and root mean squared errors (panels e and f) for seasonal mean precipitation. Here, spatial means that the statistic were performed considering all grid cells in a region. Panels g and h show the normalized inter‐annual standard deviation of area mean precipitation. Precipitation from the observational data sets (left block in each panel) and modelled precipitation (right block; M‐RCM shows results for the mean model) are compared to the precipitation of the RDs. Results for DJF/JJA are shown in the left/right panels. Hatched boxes show model results that are outside the observational uncertainties.

Observational uncertainties are also present in spatial patterns and variability, RMSE, and inter‐annual variabilities. In Figure 13(a)–(f), DJF and JJA climate mean precipitation fields from the observational data sets and the climate models are compared to the RDs. In Figure 13(g)–(h), the variability in the seasonal mean time series are compared. Models with statistical values lower/higher (in case of correlation coefficients/RMSE) or lower or higher (in case of standard deviations and inter‐annual variability) than any value in the observational ensemble are considered to be outside of the observational uncertainty. Spatial correlation coefficients of mean precipitation during DJF are high in all observational data sets in Norway, Sweden, Spain, and the UK (panel a left), while larger differences exist in the Alps, France, and Germany. All simulations show correlation coefficients within the observational uncertainties (panel a right; except the REMO run in Norway). In JJA (panel b) the observed correlation coefficients tend to agree better than in DJF (except for Norway and Sweden). Most models have higher correlation coefficients than in DJF (except in Norway and Sweden). Only the ARPEGE simulation shows correlation coefficients that are predominantly outside of the observational uncertainties.

Spatial standard deviations divided by the standard deviation of the RDs (standard deviation over all grid cells in a region) of seasonal mean precipitation are within a range of −60–20% in DJF (panel c). The RDs have always a higher standard deviation than the other observations (except CRU in the UK) while ERA‐Interim always has lower values. Most RCMs are able to capture the observed spatial standard deviations in Norway, Sweden, Spain, and the UK while they significantly overestimate (up to 200%) the variability in the other regions. In JJA, observational uncertainties are similar to DJF and vary between −60 and 35% (panel d). As in DJF, the RDs have predominantly higher spatial variabilities than the other data sets. The RCMs predominantly overestimate the spatial standard deviations in the Carpathians and France, while they commonly underestimate it in Norway and Sweden. In the Alps, the Carpathians, and France more than four RCM simulations have variabilities that are outside the observational uncertainties. These are the same areas as in DJF except for Germany.

Observational uncertainties in the spatial average RMSE of seasonal mean precipitation during DJF are largest in Norway (larger 1.9 mm day^− 1^) and smallest in the Carpathians (the analysis of relative errors leads to different results). The GPCP data set shows overall the largest RMSE values, which is likely related to its undercatch correction and the coarse grid spacing. The RCMs are predominantly within the observational uncertainties of the RMSE (except for three models in the Alps) even though they show large RMSE values in Norway (*cf* Figure 10). Only the RCA simulation has predominant significant RMSE errors. During JJA (panel f) observed RMSE differences are smaller than in DJF and the modelled RMSE are often exceeding the observational uncertainties except for Norway and Sweden. In Norway, the models have a smaller RMSE compared to the RD than any of the other observations (except for ERA‐Interim). Especially, notable is the outstanding performance of the RACMO model.

Finally, the observed normalized inter‐annual variability (standard deviation of seasonal mean, region average precipitation divided by the standard deviation of the RDs) lies within −55–35% during DJF (panel g). The RDs have a higher inter‐annual variability than the other observations except for the GPCP data set. Most models overestimate the inter‐annual variability in the Alps and the Carpathians and underestimate it in Norway, Spain, and the UK. Predominantly significant differences are found in Sweden, the Alps, and the Carpathians. In JJA, the observational uncertainties are smaller than in DJF (−40–15%; panel h). Except for Norway and Sweden, the RCM variability is predominantly outside the observational uncertainties, whereby over and underestimations of modelled variability occur. Again the RACMO model shows an outstanding performance because its variability is within the observational uncertainty range in most regions.

Summing up, observational uncertainties in the statistics shown in Figure 13 tend to be larger in DJF than in JJA. This might be caused by precipitation undercatch errors, which are smaller in JJA, and the large investigated scales (monthly on 0.5^∘^). Precipitation uncertainties are probably larger for daily and local scales. For many statistics and regions, the performance of the majority of RCM simulations is within the observational uncertainties. Nevertheless, there are some common and significant model errors that can be identified such as the overestimation of spatial variability in DJF in four of the eight regions, or the misrepresentation of the inter‐annual and spatial variability and large RMSEs in JJA.

## 6 Discussion

In this study, we show uncertainties in gridded precipitation data sets in Europe (three daily data sets and seven coarser resolution monthly data sets) and compare them with biases from eight state‐of‐the‐art high‐resolution RCM simulations from the EURO‐CORDEX project. We show that in most European regions, the magnitude of observational uncertainties is similar to those of the RCM biases. The reasons for the observational uncertainties include, among others, differences in station densities, the application of precipitation undercatch correction, data interpolation, and the impact of technical specifications such as the defined threshold for wet days.

Our results confirm previous studies (e.g. Haylock *et al.*, 2008; Hofstra *et al.*, 2010; Rauthe *et al.*, 2013; Isotta *et al.*, 2015) that show major differences between gridded observational data sets in subregions of Europe (up to 80% of seasonal mean precipitation). One major contribution to uncertainty is the density of stations networks considered in a data set. Uncertainties in observational data sets tend to decrease in regions where all data sets have a high station density (at least one station per grid cell). This highlights the need for high station densities if regional and local scale precipitation features are of interest, especially in mountainous regions. Observational errors in regions with low station density are not easily mitigated by merging them with model information, as in the example of HMR reanalysis (see also Isotta *et al.* (2015)). Nevertheless, improvements in reanalysis‐based data sets compared to data sets that use the same station network but do not have additional information from a reanalysis can be found in mountainous regions (e.g. the Alps, the Carpathians). Additional skill of regional reanalyses and high‐resolution RDs might be present on scales smaller than the 25‐km grid spacing that is investigated in this study. In addition, regional reanalyses have the advantage that they are able to provide a large set of physically consistent parameters.

One error component, which was frequently neglected in previous observation intercomparison studies, is precipitation undercatch. This component can get dominant in high latitudes and mountainous regions due to the high fraction of precipitation falling as snow (e.g. Mohr, 2009). The GPCP and RDs of Sweden and Norway include an undercatch correction and have clearly more precipitation than most of the other data sets. However, bulk undercatch correction methods as used in the GPCP data set cannot resolve regional variabilities in the amplitude of the undercatch and tend to over correct undercatch (Schneider *et al.*, 2014) at least in flat regions.

RCMs tend to agree more with undercatch corrected data sets especially in DJF and in Northern Europe and mountainous regions during JJA. Typically the biases of RCMs are within the range of observational uncertainties although frequently one or two models in the ensemble can be considered as outliers. The following guidelines should be considered in regional climate assessments of precipitation:

- To minimize sampling errors (errors in the amount, timing, intensity, and spatial structures of precipitation) a high station density is essential. The station density is primarily determining the effective resolution (actual spatial information content) of a gridded data set that can be several times larger than its grid spacing (e.g. Isotta *et al.*, 2014). Regional reanalyses have the ability to improve the spatial representation of precipitation in data sparse regions. However, they cannot fully compensate the benefits of a high‐density station network.
- A small grid spacing can easily mislead to the assumption of a high information content, which is not true in data sparse regions. We strongly advice to investigate the station density of a gridded observational data set in the region of interest before it is used. In data sparse regions, we found median uncertainties of up to 60% of the seasonal mean precipitation. Strategies to minimize uncertainties are to only use grid cells that feature stations or to upscale the gridded data set to a coarser grid.
- Precipitation undercatch can be the dominant error source in mountainous and high latitude regions (errors of up to 80%). Data sets that include a station‐based undercatch correction (specific error characteristics of rain gauges, phase of the precipitation, exposure of the stations), such as the RD of Norway and Sweden, are rare because of missing information. Bulk undercatch correction methods, such as proposed by Legates and Willmott (1990) and used in the GPCP data set, are not able to correct errors on a local scale and might over‐ or underestimate the undercatch. A way to evaluate climate models with non‐corrected observations might be to estimate the potential undercatch of simulated precipitation based on the simulated wind speed and phase of precipitation.
- The amplitude of uncertainty in observational precipitation data sets depends on the statistics of interest. High observational uncertainties are found for regional precipitation amounts (especially for extremes), spatial structures, and short‐temporal variabilities (e.g. daily scale). Lower uncertainties are found for the shape of the annual cycle, spatial variability of climate mean fields, and the inter‐annual variability of regional mean precipitation. In general, uncertainties are increasing for decreasing spatio‐temporal scales and for increasing precipitation intensities.
- Precipitation data from surface radar or radar remote sensing products can provide valuable information about spatial precipitation structures in data sparse regions (e.g. Wagner *et al.*, 2012). However, radar derived precipitation amounts are rather unreliable. Recent work is focusing on combining spatial information from radar data with precipitation amount measurements at the surface (e.g. Velasco‐Forero *et al.*, 2009; Schiemann *et al.*, 2011; Verworn and Haberlandt, 2011), which is promising to provide more accurate precipitation data sets.
- There is not a single best observational data set for regional precipitation assessments but all data sets have their strength and weaknesses. A promising strategy is the consideration of an ensemble of observational data sets from different sources (station, satellite, or reanalysis based) such as in the approach presented in this study. It might be beneficial to exclude observational data sets that show non‐physical behavior or miss important features. A sub‐selection or weighting of observational data sets therefore depends on the investigated processes and regions of interest.

## 7 Conclusion

As models are frequently tuned on the basis of observational data, misguided model development can easily result from not taking into account observational uncertainties. For example, tuning models to observations in regions where the mean model bias strongly depends on the selected observational data set (e.g. in Norway) can deteriorate the model performance. Furthermore, our results are relevant for empirical‐statistical bias correction of model output, which is usually applied before using climate simulations in climate change impact investigations. Bias correction approaches use observations to adjust biases in model output. Usually, such approaches use one single observational data set and disregard observational uncertainty. As observational uncertainty in precipitation is of a similar size to model error in many regions, the paradoxical situation may arise, that bias corrected model output is as biased, or even more biased than the uncorrected model output. This calls for improved bias correction approaches that incorporate observational uncertainty or at least clearly mark regions where bias corrected model output cannot be regarded as reliable.

Climate prediction studies are also influenced by observational uncertainties. Tuning climate models towards observations changes the model physics and therefore impacts the climate change signal. Additionally, tuning models or bias correction of model outputs in areas exhibiting large observational uncertainties (such as Scandinavia) either removes or adds precipitation, and thus has an impact on all threshold‐based climate indices such as drought indices, extreme precipitation indices, or vegetation indices. The greatest observational uncertainties are found with respect to the number of dry days and extreme precipitation intensities. These factors are particularly relevant for regional hydrology and ecosystems modelling and can affect studies related to tourism, agriculture (Cline, 2007), transportation (Polade *et al.*, 2014), flood risk (Mudelsee *et al.*, 2003), flash floods, and debris flows (Guzzetti *et al.*, 2008; Stoffel *et al.*, 2014).

If regional and local precipitation features or extreme events are of interest, we strongly encourage to use reference data sets with a high station density background. Even though our study focuses on Europe, the results are of global relevance. Observational uncertainties might be even higher in polar or in high elevated regions, or in regions where data is relatively sparse.

Following our results, it is not possible to assess model quality, when observational uncertainties are not taken into account in many European regions. Thus, we strongly suggest to use more than one observational data set for climate model evaluation and for bias correction of climate model output. Methods for sound consideration of observational uncertainty have yet to be further developed and need to become much more widespread. This is a crucial issue because in the absence of an appropriate estimate of observational uncertainties no scientifically meaningful investigation of model quality can be achieved. There is thus considerable need for observational data sets from independent sources (e.g. stations, radars, satellites) and for corresponding and more reliable uncertainty estimates. Such uncertainty estimates need to take account for problems relating to undercatch, under‐sampling, measurement techniques, homogeneity, and interpolation.
